# A clinical observational study on the application of enhanced recovery after laparoscopic pancreaticoduodenectomy

**DOI:** 10.3389/fsurg.2022.961161

**Published:** 2022-09-27

**Authors:** Rui Liao, Jun-Cai Li, Jie Chen, Xu-Fu Wei, Xiong Yan

**Affiliations:** ^1^ Department of Hepatobiliary Surgery, The First Affiliated Hospital of Chongqing Medical University, Chongqing, China; ^2^ Department of Thoracic Surgery, The People's Hospital of Yubei District of Chongqing City, Chongqing, China; ^3^ Department of Hepatobiliary Surgery, Zigong Fourth People's Hospital, Zigong, China

**Keywords:** laparoscopic, pancreaticoduodenectomy, enhanced recovery after surgery, postoperative outcome, complication

## Abstract

**Purpose:**

The safety and feasibility of enhanced recovery after surgery (ERAS) for laparoscopic pancreaticoduodenectomy (LPD) are unclear. The aim of this retrospective clinical study was to evaluate the impact of ERAS protocols for LPD.

**Patients and methods:**

Between March 2016 and December 2018, a total of 34 consecutive patients with ERAS for LPD were prospectively enrolled and compared with 68 consecutive patients previously treated for non-ERAS after LPD during an equal time frame. The intraoperative and postoperative data were collected and comparatively analyzed.

**Results:**

The mean length of postoperative hospital stay (15.8 ± 3.4 and 23.1 ± 5.1 days, *P* < 0.001) was reduced significantly in ER group than those in non-ER group. The operation time (462.7 ± 117.0 vs. 450.9 ± 109.8 min, *P* = 0.627) and intraoperative blood loss (523.5 ± 270.0 vs. 537.5 ± 241.8 ml, *P* = 0.800) were similar in the two groups. The complications (ER: 32.4% vs. non-ER: 35.3%, *P* > 0.05) and their severities (Clavien–Dindo grade ≥3 complications, 2 vs. 5 patients; *P* = 0.783) of patients with ERAS protocols were not increased. No difference in mortality and readmission rates was found. Finally, the total medical costs ($2.1 ± 0.7 × 10^4^ and $2.3 ± 0.7 × 10^4^, *P* = 0.017) in ER group were lower than those in non-ER group.

**Conclusion:**

the ERAS is safe and effective in the perioperative period of LPD. It could effectively reduce the length of postoperative stay and medical costs, and does not increase the incidence of postoperative complications.

## Introduction

Enhanced recovery after surgery (ERAS) protocols are referred to as “fast-track surgery”, which are multimodal, evidence-based approaches to the perioperative management of patients undergoing different types of operations that are designed to reduce the surgical stress response and accelerate recovery (1). The main principle of ERAS protocols is to reduce intraoperative stress and prevent immunosuppression. Moreover, ERAS protocols aim to decrease the incidence of postoperative complications and the length of hospital stays, which in turn reduce hospitalization costs. Additionally, preventing both tumour seeding and recurrence are included in the components of ERAS protocols (2). In recent years, similar promising studies have been gradually reported for various types of surgery with important clinical benefits, especially in colorectal surgery (3, 4).

With the increased publication of ERAS guidelines to optimize patient outcomes after more complex and high-risk surgical procedures, there is growing enthusiasm and interest among healthcare professionals for studies on ERAS programmes in patients after open pancreatoduodenectomy (OPD) (5, 6). Encouragingly, most initial studies have shown the feasibility and safety of ERAS for OPD, such as early discharge (6), decreased morbidity (7), and fewer postoperative complications (8). However, laparoscopic PD (LPD) remains a challenging procedure due to its technical limitations and the need for advanced skills in laparoscopy. Of note, LPD is not recommended as a preferred surgical approach if the surgeon has not mastered the long learning curve according to some clinical observations because of higher morbidity, more severe pancreatic fistulae and post-pancreatectomy haemorrhage (9). To date, few studies have been performed regarding ERAS programmes for LPD.

In the present retrospective observational study, we primarily aimed to investigate the effect of ERAS protocols in patients after LPD, including length of postoperative hospital stay and medical costs. The secondary aims were to assess the postoperative recovery and outcomes of LPD in patients in an academic medical centre in China.

## Materials and methods

### Study population

This is a retrospective clinical study on a collected data base. Between March 2016 and December 2018, a total of 102 patients who underwent LPD were enrolled in this study according to the inclusion criteria: (1) tumors of pancreas, duodenum and lower bile duct; (2) biliary obstruction caused by benign tumors (3) all patients had complete laboratory test data; (4) no anticancer treatments before the operation; (5) curative resection of all tumor nodules; and (6) complete patient records and follow-up data. Patients who had tumour metastasis and other organ resection were not considered eligible for this study. These surgeries were performed by Dr. De-Wei Li and Dr. Xiong Yan, who are experienced laparoscopic surgical experts and have completed more than 50 cases of LPD. Among these cases, 34 patients were prospectively included in enhanced recovery (ERAS group). These patients were compared with 68 consecutive patients after LPD under the care of the same consultants previously receiving standard care during an equal time frame (non-ERCP group, non-ER, Table 1). The Clavien-Dindo classification was used to evaluate the postoperative complications (10). This study protocol conformed to the ethical guidelines of the 1975 Helsinki Declaration and has been reviewed and approved by the Ethics Review Board of the First Affiliated Hospital of Chongqing Medical University (2022–031). All patients and/or their legal guardians were required to provide informed consent to participate in this study.

**Table 1:** Patients characteristics. Clinical characteristics between two groups were compared including the following risk factors: gender, age, body mass index (BMI), Abdominal surgery history, preoperative abdominal pain, preoperative diabetes and hypertension, American Association of Anaesthesiologists (ASA) score, and Child-Pugh score as well as measurements of white blood cells (WBC), neutrophils, haemoglobin, albumin, total bilirubin (TB), direct bilirubin (DB), alanine aminotransferase (ALT), and aspartate aminotransferase (AST).

Characteristics	ER group (*n* = 34)	Non-ER group (*n* = 68)	*P* value
Gender (male)	19 (55/9%)	39 (57.4%)	1.000
Age (years)	59.3 ± 9.5	61.1 ± 9.1	0.374
BMI (kg/m^2^)	22.3 ± 1.8	22.9 ± 1.9	0.174
Abdominal surgery history (year)	10 (29.4%)	17 (25.0%)	0.641
Preoperative abdominal pain	14 (41.2%)	31 (45.6%)	0.672
Preoperative diabetes (year)	7 (20.6%)	12 (17.6%)	0.719
Preoperative hypertension (year)	12 (35.3%)	22 (32.4%)	0.766
Hemoglobin (g/L)	122.1 ± 14.53	117.6 ± 15.4	0.149
WBC (10^9^/L)	7.6 ± 2.3	7.7 ± 2.4	0.713
Neutrophil (10^9^/L)	4.4 ± 2.0	5.9 ± 6.2	0.091
Albumin (g/L)	39.7 ± 4.4	40.8 ± 4.0	0.216
TBIL (µmol/L)	135.4 ± 81.7	122.7 ± 83.1	0.465
DBIL (µmol/L)	101.1 ± 77.9	97.9 ± 73.3	0.842
ALT (U/L)	158.0 ± 130.8	136.1 ± 102.8	0.397
AST (U/L)	140.7 ± 119.6	120.0 ± 99.0	0.388
PT (s)	12.9 ± 1.5	13.0 ± 1.6	0.652
ASA score			0.337
I	16 (47.1%)	36 (53.0%)	
II	18 (52.9%)	29 (42.6%)	
III	0 (0)	3 (4.4%)	
Child-Pugh Grade			1.000
A	14 (41.2%)	27 (39.7%)	
B	20 (58.8%)	41 (60.3%)	

Abbreviations: ER, enhanced recovery after surgery; BMI, Body mass index; WBC, white blood cells; TBIL, total bilirubin; DBIL, direct bilirubin; ALT, alanine aminotransferase; AST, aspartate *aminotransferase*; PT, prothrombin time; ASA, American Society of Anesthesiologists score.

The continuous variable data were expressed as mean ± standard deviation.

### ERAS protocols

ERAS protocols were designed based on previously published ERAS guidelines (1), and we further refined and established our own standardized programmes (Table 2). Early oral intake and ambulation, active pain control, avoidance of unnecessary indwelling medical tubes, and promotion of patient autonomy were encouraged if the condition of the patients allowed. After surgery, patients went go to the observation unit and then moved back to the ward. If vital signs were unstable, the patients went to ICU postoperatively. On postoperative day (POD) 1, anti-thrombotic prophylaxis, especially VTE prophylaxis and post VTE prophylaxis were performed. From POD 1, the patients began ambulation such as mobilization out of bed for >1 h or at least 3 times. If necessary, ambulation assist equipment could be used. Appropriate glucose loading was used on the day of surgery based on the energy need of the patients. Postoperative oral intake of liquids was started on POD 1–2, and elemental diet was started on POD 2, and solids were started on POD 3–4. Intake of rice porridge was started on POD 3 in parallel with elemental diet. Postoperative fluid management was adjusted based on oral intake. The intravenous fluid infusion volume was controlled within 1000–1500 ml. Nasogastric tubes were removed on POD 1 if nasogastric output was <300 ml/day. The urinary catheter was also removed on POD 1. Intra-abdominal drains were removed on POD 3 if amylase was less than 3-fold higher than the serum level and output was <50 ml/d. Physiotherapy was started on POD 1 and continued until discharge. For postoperative prophylaxis, piperacillin/tazobactam sodium was administered intravenously on the operative day (4.5 g, three times). The compliance with ERAS and non-ERAS core elements of each patient was recorded as either 0 for non-accomplished or 1 for accomplished.

**Table 2:** Protocols of the ERAS program and traditional care for patients receiving LPD. All items involved in the protocols were list in the table including preoperative, intraoperative and post-operative 1–4 days.

	ER group	Non-ER group
Preoperative	Counseling about fast-track rehabilitation programme while admission.	Traditional informed consent.
	Intake of clear fluids up to 3 h before anesthesia and normal oral nutrition until 6 h before surgery.	Overnight fasting.
	No bowel preparation before surgery	Oral bowel preparation.
Intraoperative	Restrictive intravenous fluids	Restrictive intravenous fluids
	Perioperative antibiotic	Perioperative antibiotic
	Somatostatin	Somatostatin
	Acid suppression	Acid suppression
	Analgesia infusion pump	Analgesia infusion pump
	Postoperative vomiting prophylaxis with 5-HT receptor antagonist	
POD 1	Clear oral liquids and restricted amounts of intravenous fluids including glucose	Fasting till flatus and no restricted amounts of intravenous fluids
	Steam inhalation	Steam inhalation
	Anti-thrombotic prophylaxis	Anti-thrombotic prophylaxis
	Removal of urinary catheter	On-bed movement
	Removal of nasogastric tube if <300 ml	
	Mobilization out of bed for *>*1 h or at least 3 times	
	Physiotherapy	
POD 2	Oral elemental diet	Removal of urinary catheter after intermittent clipping
	Enhanced mobilization out of bed for *>*2 h or at least 6 times	Removal of nasogastric tube after flatus
POD 3	Oral semisolid diet	Oral intake of elemental diet depending on patient's progress including flatus
	Stop antibiotics	Removal of urinary catheter after intermittent clipping
	Removal of intra-abdominal drains if drain amylase less than 3-fold serum and 50 ml/d production	Bedside sitting and standing according to patient's ability
	Enhanced mobilization out of bed for *>*4 h	
POD 4	Gradual transfer to regular diet	Oral liquids
	All medication stopped except oral proton pump inhibitors and multivitamin	Stop antibiotics according to the inflammatory index
		Removal of intra-abdominal drains if drain amylase less than 3-fold serum and 50 ml/d production
		Untargeted mobilization
Discharge criteria	Normal body temperature	Normal body temperature
	Ability to take solid foods without vomiting even only 1–2 times/day	Regular diet like the time before illness
	Adequate mobilization	Adequate mobilization
	No abdominal pain or controlled abdominal pain by oral pain medications (Tramadol 50mg, no more than twice per day, $0.32/tablet)	No abdominal pain
	No obvious abnormalities in laboratory test	No obvious abnormalities in laboratory test

Abbreviations: ERAS, enhanced recovery after surgery; LPD, laparoscopic pancreaticoduodenectomy; 5-HT, 5-hydroxytryptamine; POD post-operation day.

### Surgical procedures

The standard surgical procedures were performed in all patients, including gastrointestinal anastomosis and the pancreaticojejunostomy (anastomosis of pancreatic duct mucosa and jejunum mucosa). The intra-abdominal drains were routinely placed at the site of the bilioenteric anastomosis and pancreaticojejunostomy.

### Statistical analysis

SPSS 24.0 statistical software was used to analyse the data. The continuous variable data are shown as the mean ± standard deviation. Continuous variables were compared using Student's *t*-tests with a normal distribution or nonparametric Mann–Whitney *U*-tests with an irregular distribution. Count data were analysed using *x*^2^ test, Fisher's exact test and the rank sum test. A *P* value <0.05 was considered statistically significant.

## Results

### Patient characteristics

Clinical characteristics between two groups were compared using the following risk factors: gender, age, body mass index (BMI), American Association of Anaesthesiologists (ASA) score, and Child-Pugh score as well as measurements of white blood cells (WBC), neutrophils, haemoglobin, albumin, total bilirubin (TB), direct bilirubin (DB), alanine aminotransferase (ALT), and aspartate aminotransferase (AST) (Table 1). This study included 58 men (56.9%) and 44 women (43.1%) with an average age of 60.5 ± 9.2 years. The two groups were similar in terms of demographics and surgical characteristics (all *P* > 0.05). There was no difference in mean age, BMI, ASA score and Child-Pugh score between the ER and non-ER groups (*P* > 0.05). Common laboratory tests, including WBC, TB, DB, AST, ALT and albumin, did not differ significantly between the two groups (*P* > 0.05).

### Operative outcomes

There were no statistically significant differences were observed between the ER and non-ER groups in duration of surgery, intraoperative blood loss and transfusion, which are presented in Table 3. In the ER group, the mean duration of surgery was 462.7 (300–720) min, the operative blood loss was 523.5 (250–1200) ml, and 8.8% of patients received an intraoperative transfusion. However, in the non-ER group, the mean duration of surgery, operative blood loss and proportion of patients who received an intraoperative transfusion were 450.9 (300–760) min (*P* = 0.627), 537.5 (250–1500) ml (*P* = 0.800) and 11.8% (*P* = 0.746), respectively. The intravenous fluid infusion rate was 4.0 ml/kg/h. Usually, the ratio of crystalloid to colloid was from 2:1 to 3:1. The ER group had significantly lower total fluid and crystalloid volume than non-ER group (*P* < 0.01). There was no significant difference in the colloid volume between two groups. All patients used analgesic infusion pump in both groups.

**Table 3:** Demographics and perioperative variables in patients. 17 perioperative variables were used to compare between two groups including duration of surgery, intraoperative blood loss, patients transfused, intraoperative biliary drains, pathologic diagnosis, pancreatic cancer, cholangiocarcinoma, duodenal adenocarcinoma, benign lesion, nasogastric tube removal time urinary catheter removal time, intra-abdominal drains removal time, first bowel gas time, first diet time, off-bed activity time, postoperative stay and total medical cost.

Variables	ER group (*n* = 34)	Non-ER group (*n* = 68)	*P* value
Duration of surgery (min)	462.7 ± 117.0	450.9 ± 109.8	0.627
Intraoperative blood loss (ml)	523.5 ± 270.0	537.5 ± 241.8	0.800
Patients transfused (year)	3 (8.8%)	8 (11.8%)	0.746
Intraoperative biliary drains (year)	29 (85.3%)	48 (70.6%)	0.143
Pathologic diagnosis			
Pancreatic cancer	18 (52.9%)	43 (63.2%)	0.393
Cholangiocarcinoma	8 (23.5%)	12 (17.6%)	0.598
Duodenal adenocarcinoma	5 (14.7%)	7 (10.3%)	0.528
Benign lesion	3 (8.8%)	6 (8.8%)	1.000
Nasogastric tube removal time (days)	1.8 ± 0.9	3.6 ± 1.1	**<0** **.** **001**
Urinary catheter removal time (days)	2.1 ± 1.0	3.8 ± 1.1	**<0** **.** **001**
Intra-abdominal drains removal time (days)	4.2 ± 1.2	7.2 ± 2.6	**<0** **.** **001**
First bowel gas time (days)	2.7 ± 0.9	4.0 ± 0.9	**<0** **.** **001**
First diet time (days)	2.0 ± 0.6	3.5 ± 0.7	**<0** **.** **001**
Off-bed activity time (days)	2.3 ± 0.7	4.2 ± 0.9	**<0** **.** **001**
Postoperative stay (days)	15.8 ± 3.4	23.1 ± 5.1	**<0** **.** **001**
Total medical cost (¥10^3^)	14.3 ± 2.8	15.8 ± 2.9	**0** **.** **017**

Abbreviations: ER, enhanced recovery after surgery. The continuous variable data were expressed as mean ± standard deviation.

#### Post-operative outcomes

Post-operative pathologic diagnosis found 3 and 6 benign lesions in ER group and non-ER group, respectively (*P* = 1.000). Others were tumors including pancreatic cancer, cholangiocarcinoma and duodenal adenocarcinoma (*P* > 0.05). Compared with non-ER groups, patients with ERAS spent shorter time in nasogastric tube remove, urinary catheter remove, intra-abdominal drains remove, first bowel gas time, first diet time and off-bed activity (all *P* < 0.01). The mean length of postoperative hospital stay was 15.8 ± 3.4 and 23.1 ± 5.1 days (*P* < 0.001) in the ER group and non-ER group, respectively.

### Postoperative complications

The complications included pancreatic or biliary fistula, haemorrhage, abdominal or pulmonary infection, unplanned reoperation, mortality rate and readmission within 30 days of the operation (Table 4). The overall complication rate was 32.4% (11 of 34 patients) in the ER group compared with 35.3% (24 of 68) in the non-ER group (*P* = 0.281). According to the clinical effect, postoperative pancreatic fistula has three grades (grade A, B and C) based primarily on the International Study Group on Pancreatic Fistula (ISGPF) (11). There were 7 and 14 patients with a grade A pancreatic fistula happened in the ER group and non-ER group, respectively. 3 and 5 patients in the ER group and non-ER groups developed a grade B pancreatic fistula after surgery, respectively. These patients were healed after treatment with abdominal puncture and B-ultrasound guided-drainage. There was no significant difference in the pancreatic fistula ratio between the two groups (*P* = 0.903). There was one patient with biliary fistula, delayed gastric emptying and pleural effusions or ascites in each group (*P* = 0.615), respectively. One patient with postoperative haemorrhage in non-ER group underwent a second surgery. Two patients with postoperative haemorrhage in ER group received blood transfusion and hemostasis treatments other than surgery (*P* = 0.216). In ER group, there was no patient with abdominal, pulmonary or wound infection, thrombosis, unplanned reoperation and dead. Correspondingly in patients with non-ER, there was one patient producing abdominal, or wound infection, thrombosis and unplanned reoperation, respectively (*P* = 0.480). One patient was dead (*P* = 0.480), and three patients underwent pulmonary infection (*P* = 0.216). Except one unplanned reoperation, the other postoperative complications were treated conservatively. One patient with ER chose readmission in 30 days due to ascites. According to the Clavien–Dindo grade (10), there was no significant difference in grade ≥ 3 complications between ER group and non-ER group (2 vs. 5 patients; *P* = 0.783). There was no difference in postoperative complications between the patients after LPD performed by Dr. Li and Dr. Yan. The median duration of postoperative fluid management was 9.5 days (range: 7.5–14 days) in the ER group and 14 days (range: 12.5–22 days) in the non-ER group (*P* < 0.01).

**Table 4:** Surgical complications. 12 Surgical complications were used to compare between two groups including pancreatic fistula, biliary fistula, postopreative hemorrhage, abdominal infection, pulmonary infection, wound infection, delayed gastric emptying, pleural effusions or ascites, thrombosis, unplanned reoperation, mortality, readmission in 30 days.

Types	ER group (*n* = 34)	Non-ER group (*n* = 68)	*P* value
Pancreatic fistula, *n* (%)			0.903
Grade A	7 (20.6%)	14 (20.6%)	
Grade B	3 (8.8%)	5 (7.4%)	
Biliary fistula, *n* (%)	1 (2.9%)	1 (1.5%)	0.615
Postopreative hemorrhage, *n* (%)	2 (5.9%)	1 (1.5%)	0.216
Abdominal infection, *n* (%)	0 (0)	1 (1.5%)	0.480
Pulmonary infection, *n* (%)	0 (0)	3 (4.4%)	0.216
Wound infection, *n* (%)	0 (0)	1 (1.5%)	0.480
Delayed gastric emptying, *n* (%)	1 (2.9%)	1 (1.5%)	0.615
Pleural effusions or ascites, *n* (%)	1 (2.9%)	1 (1.5%)	0.615
Thrombosis, *n* (%)	0 (0)	1 (1.5%)	0.480
Unplanned reoperation	0 (0)	1 (1.5%)	0.480
Mortality	0 (0)	1 (1.5%)	0.480
Readmission in 30 days, *n* (%)	1 (2.9%)	0 (0)	0.157
Clavien–Dindo grades			
No	23 (67.6%)	44 (64.7%)	
1–2	9 (26.5%)	19 (14.7%)	
3a	1 (2.9%)	1 (1.5%)	
3b	0 (0)	1 (1.5%)	
4a	1 (2.9%)	1 (1.5%)	
4b	0 (0)	1 (1.5%)	
5	0 (0)	1 (1.5%)	
Grad ≥3	2 (18.2%)	5 (20.8%)	0.782

### Cost analysis

The mean total medical costs per patient were $2.1 ± 0.7 × 10^4^ and $2.3 ± 0.7 × 10^4^ in ER group were lower than those in non-ER group. in the ER and non-ER groups, respectively (*P *= 0.017). Postoperative mean costs per patient were $2.0 × 10^4^ in the ER group and $2.2 × 10^4^ in the non-ER group (*P* = 0.019). ERAS was significantly associated with lower costs for medication, housing, nursing care and disposable material costs (*P* < 0.05) in the ER group compared with the non-ER group. The surgical and anesthetic costs were similar in the two groups (*P* > 0.05). In the enhanced recovery group, these patients had some extra radiological examinations or laboratory examinations to prevent accidental complications due to the enhanced recovery. Other laboratory and radiologic examination costs had no significant difference (*P* > 0.05).

### Compliance with ERAS core elements

The recovery indicators for patients with ERAS or non-ER were comparatively analysed. The retention time of nasogastric tubes, urinary catheters and intra-abdominal drains in the ER group were significantly shorter than that in the non-ER group (*P* < 0.001). In addition, oral elemental diet and targeted mobilization after surgery in the ER group also occurred much earlier than that those in the non-ER group (*P* < 0.001) (Table 5**)**. In addition, there were several patients deviating from the ERAS protocols. The nasogastric tube was removed on POD 1 in 26 (76.5%) patients, but four patients (11.8%) could not start oral intake of an elemental diet on POD 2. The overall compliance rates to protocol were 89.2% in ER group and 57.4.8% in non-ER group (*P* < 0.01), respectively. As shown in Table 5, early removal of urinary catheter and intra-abdominal drains were more difficult to follow in both groups. Compare with ER group, it was not easy to perfome vomiting prophylaxis, early removal of nasogastric tube and early oral elemental diet in patients with non-ER (*P* < 0.001).

**Table 5:** Compliance with factors of ERAS protocols. 15 compliance factors of ERAS protocols were used to compare between two groups including preoperative counseling, no bowel preparation, antimicrobial prophylaxis, restrictive intravenous fluids, somatostatin, acid suppression, analgesia pump, vomiting prophylaxis, early removal of urinary catheter, early removal of nasogastric tube, early removal of intra-abdominal drains, early oral elemental diet, anti-thrombotic prophylaxis, steam inhalation, targeted mobilization.

Factors	ER group (*n* = 34)	Non-ER group (*n* = 68)	*P* value
Preoperative counseling	34 (100%)	0 (0)	**<0** **.** **001**
No bowel preparation	30 (88.2%)	2 (2.9%)	**<0** **.** **001**
Antimicrobial prophylaxis	34 (100%)	68 (100%)	–
Restrictive intravenous fluids	34 (100%)	68 (100%)	–
Somatostatin	34 (100%)	68 (100%)	–
Acid suppression	34 (100%)	68 (100%)	–
Analgesia pump	33 (97.1%)	65 (95.6%)	1.000
Vomiting prophylaxis	29 (85.3%)	10 (14.7%)	**<0** **.** **001**
Early removal of urinary catheter	21 (61.8%)	12 (17.6%)	**<0** **.** **001**
Early removal of nasogastric tube	26 (76.5%)	13 (19.1%)	**<0** **.** **001**
Early removal of intra-abdominal drains	17 (50.0%)	9 (13.2%)	**<0** **.** **001**
Early oral elemental diet	30 (88.2%)	8 (11.8%)	**<0** **.** **001**
Anti-thrombotic prophylaxis	34 (100%)	64 (94.1%)	0.298
Steam inhalation	32 (94.1%)	59 (86.8%)	0.328
Targeted mobilization	33 (97.1%)	45 (66.2%)	**<0** **.** **001**

Abbreviations: ERAS, enhanced recovery after surgery.

## Discussion

To the best of our knowledge, this is the first clinical observational study in China to investigate the feasibility and safety of ERAS protocols in patients after LPD. The present study suggests that ERAS programmes may have benefits in facilitating earlier patient recovery, which included earlier first bowel gas time, first diet time, and off-bed activity time; shorter drainage tube retention time; shorter postoperative stay; and lower medical costs. Meanwhile, there was no significant difference in most postoperative complication rates and 30-day readmissions between the two treatments.

Compared to OPD, LPD is a less invasive surgical procedure with higher risk that requires advanced laparoscopic skills for senior surgeons. The incidence of postoperative complications of LPD is still as high as 30%–50%, even when performed by an expert surgeon. Thus, it seems that ERAS programmes for LPD might not be widely accepted. In fact, implementation of ERAS is now expanding across a wide range of complex laparoscopic surgical procedures and specialties, such as colorectal resection (12), liver resection (13), and gynaecologic oncology (14), and the benefits of ERAS have been well proven in these surgeries. For the first time, our observational results demonstrated the safety and efficiency of implementing ERAS protocols for LPD. However, RCTs are required to provide further evidence about ERAS protocols for LPD.

In this study, the postoperative stay was decreased significantly in ER group. This may be clinically important because early oral elemental diet and targeted mobilization lead to fast recovery and increased immunity of patients after surgery, and subsequently reducing the length of hospital stay. In the ER group, earlier first diet and off-bed activity after surgery were encouraged for patients following LPD, which may have resulted in earlier bowel gas. Currently, increasing studies have shown that long-term fasting may lead to slower recovery of intestinal peristalsis (15) and increased risk of metabolic disorders (16), which is not conducive to patient recovery. Early enteral feeding can reduce postoperative infections and shorten postoperative hospital stays (17). Feeding proximally to the anastomosis does not increase the risk of bowel anastomosis (18). Balzano et al. showed that early feeding did not increase the incidence of pancreatic fistula after surgery but could reduce the incidence of gastric emptying (19). Considering the complexity of LPD, in our protocols, the patients turned over or moved their limbs on the bed on POD 1, then moved legs on the bedside, and finally got out of bed for small-scale exercise. We thought that the premise for early ambulation was effective analgesia and early removal of various drainage tubes.

The shorter retention time of urinary catheters, nasogastric tubes and intra-abdominal drains could reduce some related postoperative complications. Indwelling urinary catheters can increase the risk of postoperative lung and urinary tract infections (20), and indwelling nasogastric tubes contribute to a high risk of lung infection in patients (21), which further delays patient recovery. Moreover, a multicentre RCT showed that the absence of an intra-abdominal drain after OPD significantly increased the incidence of complications and mortality by 4 fold (22). Therefore, this practice is reasonable to implement in patients with intra-abdominal drains following LPD. However, this study also suggests that early drain removal based on the amount of postoperative drainage is acceptable in patients undergoing LPD.

Our data suggest the efficiency of ERAS programmes for decreasing postoperative complications and morbidity, which is consistent with a previous RCT on ERAS protocols for OPD (7). Moreover, a recent retrospective study found that lower pre-albumin level, higher ASA score and longer operative time were independent risk factors for failure of early recovery from OPD and increased complications of ERAS for OPD (5). Of note, we found that more patients had pulmonary infections in the non-ER group compared with the ER group. This result may be associated with the early ambulation of ERAS protocols, which could promote expectoration and immunity of patients and decrease the opportunity for pulmonary infection.

ERAS pathways showed reduced medical costs with LPD. Compared to non-ER, surgical and anaesthetic costs were not different, but the costs of wards and beds, laboratory and radiologic examinations, and medications were decreased significantly, which resulted in lower total medical cost. Other reasons for reduced medical costs may be attributed to the shorter postoperative hospitalization time and less postoperative complications, with no increase in the readmission rate. Consequently, the patients could benefit from the reduced healthcare costs of LPD *via* ERAS programs.

Another important finding was that ERAS programmes did not increase the risk of postoperative haemorrhage and biliary or pancreatic fistula. Given the technically challenging nature of the LPD procedure, it requires higher laparoscopic skill with regard to accurate needle handling to prevent suture tangling in the biliary/pancreatic ducts and intestinal/pancreatic tissues. In our study, the two surgeons were experienced laparoscopic surgical experts and completed the initial learning curve, which could help to avoid technical bias. Moreover, we do not support the recommendation for routine preoperative endoscopic nasal biliary drainage for patients undergoing LPD.

The main limitations of our study are the nature of the retrospective study and the small sample in a single medical centre. We have not compared the surgical effects between LPD and OPD during the same period. To date, there is no widely accepted guideline or recommendation for ERAS programmes for LPD. Therefore, all the basic components of the “fast-track” or ERAS programmes for LPD in our study are based on other protocols for OPD in other different laparoscopic surgical specialties. Some factors would produce some differences in the implementation of ERAS between LPD and OPD, such as the influences of surgical wound size, carbon dioxide pneumoperitoneum pressure, patient psychology status induced by different surgical methods and diet time. However, according to our results, the majority of OPD protocols could be implemented in the ERAS of LPD. A different selection of LPD patient population mainly comes from the requirements of laparoscopic surgery. Moreover, our results need to be validated by additional future studies or stratified analysis. Furthermore, compared to the west countries, the mean length of postoperative hospital stay was still longer in both groups. The difference may come from the eastern traditional culture of health care requiring long-term postoperative recuperation. On the other hand, Chinese medical insurance policy does not strictly control the length of the hospital stay and many Chinese patients did not strictly follow the doctor's discharge schedule. We believe it could be further shortened if Chinese patients are more able to follow the surgeons' guidance. In addition, the introduction of laparoscopic procedure perhaps led to clinicians challenging their traditional viewpoints of postoperative care which has been aided by the ERAS groups research. Therefore, a more evidence based postoperative care program should be introduced in the future.

In summary, ERAS protocols for LPD can effectively reduce the length of postoperative stay and medical costs and do not increase the incidence of postoperative complications. However, individualized ERAS measures for LPD should be formulated in light of clinical practice and previous studies. In future studies, more intensive multimodal approaches should be optimized and improved to maximize patient benefit.

## Data Availability

The original contributions presented in the study are included in the article/Supplementary Material, further inquiries can be directed to the corresponding author/s.
